# Effectiveness of Different Application Modalities on the Bond Performance of Four Polymeric Adhesive Systems to Dentin

**DOI:** 10.3390/polym15193924

**Published:** 2023-09-28

**Authors:** Rim Bourgi, Louis Hardan, Carlos Enrique Cuevas-Suárez, Walter Devoto, Cynthia Kassis, Khalil Kharma, Ryan Harouny, Tarek Ashi, Davide Mancino, Naji Kharouf, Youssef Haikel

**Affiliations:** 1 Department of Restorative Dentistry, School of Dentistry, Saint-Joseph University, Beirut 1107 2180, Lebanon; rim.bourgi@net.usj.edu.lb (R.B.); louis.hardan@usj.edu.lb (L.H.); cynthia.kassis@usj.edu.lb (C.K.); khalil.kharma@usj.edu.lb (K.K.); ryaneliott.harouny@net.usj.edu.lb (R.H.); 2 Department of Biomaterials and Bioengineering, INSERM UMR_S 1121, University of Strasbourg, 67000 Strasbourg, France; tarekachi@live.com (T.A.); mancino@unistra.fr (D.M.); youssef.haikel@unistra.fr (Y.H.); 3 Dental Materials Laboratory, Academic Area of Dentistry, Autonomous University of Hidalgo State, San Agustín Tlaxiaca 42160, Mexico; cecuevas@uaeh.edu.mx; 4 Independent Researcher, 16030 Sestri Levante, Italy; walter@walterdevoto.com; 5 Craniofacial Research Laboratory, Division of Biomaterials, School of Dentistry, Saint-Joseph University, Beirut 1107 2180, Lebanon; 6 Department of Endodontics and Conservative Dentistry, Faculty of Dental Medicine, University of Strasbourg, 67000 Strasbourg, France; 7 Pôle de Médecine et Chirurgie Bucco-Dentaire, Hôpital Civil, Hôpitaux Universitaire de Strasbourg, 67000 Strasbourg, France

**Keywords:** application techniques, bond strength, microtensile bond strength, resin-dentin interface, universal adhesive

## Abstract

One of the major goals of adhesive dentistry is to improve the interaction of the already-existing adhesives with different substrates by using different application techniques. Thus, the objective of the present in vitro study was to assess the bond performance of four adhesive systems, Prime&Bond Universal (PBU), Clearfil SE Bond (CSE), OptiBond Universal (OBU), and OptiBond FL (OBFL), to dentin using various application modes: passive application (PA), active application (AA), Compo-Vibes modified application (CVM), and Compo-Vibes application (CV). Eighty extracted human molars were allocated into four groups based on the application modalities tested. The micro-tensile bond strength as well as fracture mode were tested in accordance with ISO/TS 11.405 after 24 h and 6 months of aging. Adhesive contact angle (CA) and scanning electron microscope analysis were also performed (*n* = 3). Statistical tests were performed with α = 0.05. After 24 h, a significant difference with a higher bond strength value was found for PBU in the AA modality and for CSE in the CVM modality (*p* < 0.05). However, no significant difference was shown between the techniques used among the other adhesives (OBFL and OBU). Moreover, at 24 h, only the PA demonstrated significant differences between the tested materials (*p* < 0.05). After 6 months, CSE, PBU, and OBU demonstrated significant differences between the techniques (*p* < 0.05), with a higher bond strength for CSE in AA and CVM modalities, for PBU in AA modality, and for OBU in AA and PA modalities. No significant differences were found between the techniques used among the OBFL (*p* > 0.05). In addition, only the CVM technique demonstrated significant differences between the tested materials after 6 months. CV and CVM showed a decreased value after aging for CSE and PBU, respectively. However, all the modalities decreased for OBU and OBFL after aging. All the adhesives showed marked resin infiltration into dentinal tubules in AA among all the modalities tested. Both universal adhesive systems (OBU and PBU) demonstrated statistically lower CA when compared to the other systems (CSE and OBFL) (*p* < 0.05) when applied in the PA mode. Concerning the AA mode, only CSE and OBFL were tested. The AA demonstrated lower CA values compared to the same adhesives in PA (*p* < 0.05). It could be concluded that the bond strength could be influenced by both materials and application techniques. It seems that the AA technique could be recommended as a gold standard for the application of an adhesive system to dentin. Plus, the CV and CVM modalities after 6 months of aging were considered stable for PBU and CSE, respectively. Consequently, the performance of these adhesive systems might vary when applied to other modalities. Future studies are needed to test this hypothesis.

## 1. Introduction

Regardless of the strategy used in adhesion procedures, including etch-and-rinse (ER) or self-etch (SE) adhesives, dentinal bond quality necessitates the creation of a structure called the hybrid layer (HL), which is comprised of a demineralized layer of collagen fibrils reinforced by the solvated resin matrix [1]. The stability of the HL ultimately depends on each component’s resistance to degradation, regardless of the adhesive layer thickness and/or tags’ length [2]. Efforts to reach a durable resin-dentin bond have been made, embracing the mechanisms slowing down or halting the HL disorganization and degradation, perfecting the dental adhesive system manufacturing process, as well as evolving modalities for the protection of the HL [3].

Among the ER adhesives, OptiBond FL (OBFL, Kerr Co., Orange, CA, USA), a three-step ER adhesive, has been widely considered one of the gold standards of multiple-bottle systems [4]. This adhesive incorporates a highly hydrophobic bonding agent, which includes glycero-phosphate dimethacrylate (GPDM), which can react chemically with hydroxyapatite in the etched and primed enamel and dentin substrate to enhance bond resilience [5]. Subsequently, SE systems were initiated, abolishing the application of phosphoric acid and the subsequent rinsing step [6]. Among the SE adhesives, Clearfil SE Bond (CSE, Kuraray Noritake Dental, Tokyo, Japan) is documented as a gold standard in this category; the primer and the adhesive of this system include 10-methacryloyloxydecyl dihydrogen phosphate (10-MDP), which reacts with calcium to generate a strong and stable bond, thus forming nanolayers of 10-MDP-calcium [7].

A conventional view of dental bonding by means of a faster application was recently introduced to the market [8]. A less sensitive technique with numerous application options is feasible for clinicians nowadays with the arrival of universal adhesives (UAs) [9]. These systems have three different modes of application: ER, SE, and selective-etch. They can also adhere to multiple substrates, including resin composites, zirconia, silica-based ceramics, and metals [10]. Evidence from a previous systematic review and meta-analysis suggested that the use of the SE adhesion approach with this new type of adhesive appears to be an optimal option that enhances the bond strength to the dentin structure [11].

Accordingly, one of the major goals of adhesive dentistry is guided by two main tendencies: the development of the next generation of dental adhesives based on UAs and overcoming their shortcomings. The second tendency is related to the improvement of the interaction of the already-existing adhesives with the different substrates by using different application techniques [12]. Thus, numerous methods have enhanced the bonding effectiveness of adhesive systems while sustaining their versatility and multi-functional characteristics; these include the application of an extra hydrophobic resin coat after adhesive application and a double-layer application technique (two layers and more). Furthermore, active application (AA) along with solvent evaporation for longer than 10 s may be recommended for improving the bond strength of the materials [12,13]. Moreover, using a warm-air stream on the primer or the adhesive system is recommended for better bonding [14].

Standardized dentin bond strength assessments assist in quantifying the dentin bond performance of dental adhesives, comparing dentinal bond strength values between different products and conditions, screening bonding capability, and understanding the mechanism of bonding from a mechanical perspective. Yet, the nature of the reactions that occur between adhesives and the dentin substrate remains unclear, and because of that, qualitative and morphological evaluations are compulsory. Scanning electron microscopy (SEM) and transmission electron microscopy are examples of the aforementioned tests employed in bonding mechanism research [15]. However, the predominant technique is SEM due to its user friendliness. SEM can be obtained either in secondary mode [16] or in backscattered mode [17].

Bonding efficiently to organic and wet dentin is a baffling task in the era of adhesive dentistry. Sano et al., [18] stated that the micro-tensile bond strength (μTBS) could be better than the shear bond strength test to consider the bond strength of an adhesive system, while another report described that the significant factor for bond strength is the bonding agent used, irrespective of the testing exemplary used [19]. For optimal adhesion, an adhesive must completely “wet” the surface to be bonded [20]. “Wetting” means that the adhesive flows and covers a surface to maximize the contact surface and the forces of attraction between the adhesive and this bonding surface. Wettability is generally based on the measurement of contact angle (CA) as primary data, which indicates the degree of wetting when a solid and a liquid interact. A small CA lower than 90° (<90°) corresponds to high wettability, while a large CA (>90°) corresponds to low wettability. The CA is defined as the angle formed by the intersection of the liquid adhesive-solid dentin interface [21].

Recently, a new device called Compo-Vibes (Smile Line, St-Imier, Switzerland) was launched for easier, faster, and more reliable composite modeling. This device generates micro-vibrations of 0.158 kHz or 158 Hz with a tolerance of approximately +/−15% for more precise composite applications, made possible by different tips. Additionally, a special brush, which has multiple functions, was attached to this device and used in this study for bonding applications. Till now, to the best of researcher knowledge, there have been no studies that have simultaneously compared the bonding effectiveness of four application modalities: passive application (PA, applying the adhesive without any agitation), AA (applying the adhesive with active agitation), micro-vibration with Compo-Vibes (CV, Smile Line, St-Imier, Switzerland) (applying the adhesive with the help of a Compo-Vibes (Smile Line, St-Imier, Switzerland) tip used as a brush for bonding), or Compo-Vibes (Smile Line, St-Imier, Switzerland) modified application (CVM) using a micro-brush. Hence, this article aims to assess the bonding performance of four adhesive systems to dentin using various application modes: PA, AA, CV, and CVM. According to the null hypothesis, the application method has no effect on the: (i) immediate and long-term bond strength of the adhesive systems to dentin following the four modalities; and (ii) dentinal wettability following AA and PA.

## 2. Materials and Methods

### 2.1. Materials

In this study, the μTBS of four adhesive systems were analyzed considering different application modalities: (1) PA, applying the adhesive without any agitation; (2) AA, applying the adhesive with active agitation; and (3) micro-vibration with Compo-Vibes (Smile Line, St-Imier, Switzerland), applying the adhesive with the help of a Compo-Vibes (Smile Line, St-Imier, Switzerland) brush for bonding (CV) or a (4) micro-brush (CVM). Two UAs, Prime&Bond Universal (PBU, Dentsply DeTrey GmbH, Konstanz, Germany) and OptiBond Universal (OBU, Kerr Co., Orange, CA, USA), one three-step ER adhesive, OBFL, and one two-step SE adhesive, CSE, were evaluated. The sample size was estimated based on previous literature that evaluated the bond strength of an adhesive system using different application modalities in a comparative study design with 4 independent groups [22]. Using an α of 0.05, a power of 80%, and a two-sided test, the minimal sample size was 5 specimens in each group in order to detect a difference of 5 MPa among the tested groups.

The composition of the adhesive systems evaluated in this study is described in Table 1.

**Figure 1:**
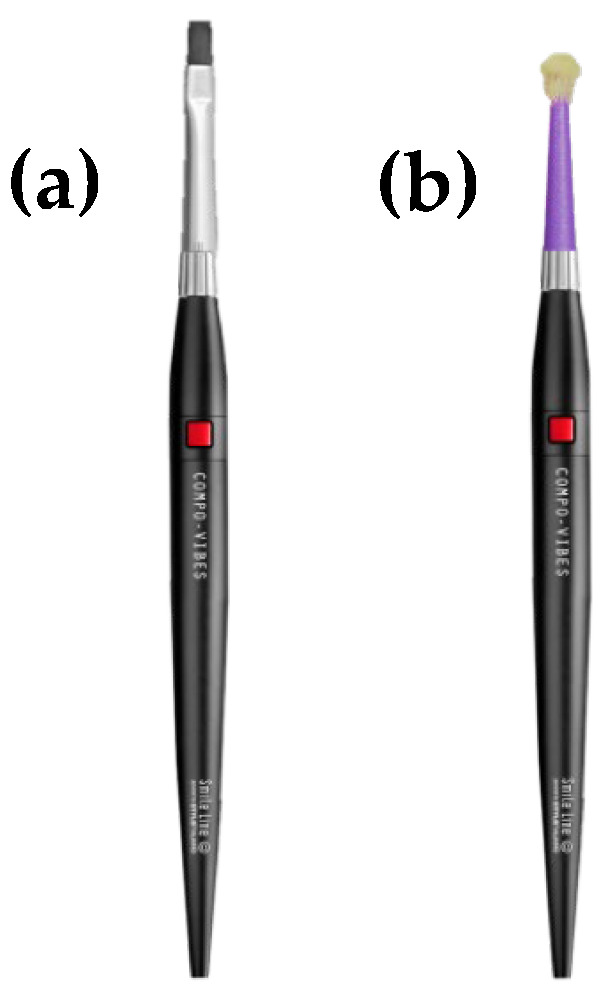
Representative images of the Compo-Vibes (Smile Line, St-Imier, Switzerland) instrument (an application modality could be possible with the help of the Compo-Vibes (Smile Line, St-Imier, Switzerland) brush in (**a**) used for bonding in this study or by using a micro-brush in (**b**)).

Four groups based on the application modalities (PA, AA, CV, and CVM) were used. For PA, all adhesives tested were applied for 20 s and left undisturbed; for AA, all adhesives tested were applied with active agitation for 20 s. Further, for micro-vibration with CV and CVM, all adhesives tested were applied for 20 s with the Compo-Vibes (Smile Line, St-Imier, Switzerland) instrument (this could be possible with the help of the Compo-Vibes (Smile Line, St-Imier, Switzerland) brush used for bonding in this study (Figure 1a) or by using a micro-brush (Figure 1b)). It is important to note that for the CVM modality, a micro-brush was cut as shown in Figure 1b. Furthermore, the red button on the handle allowed for micro-vibration of both CV and CVM modalities.

### 2.2. Bonding and Sample Preparations

Eighty sound human molars extracted for periodontal and orthodontic reasons were collected, cleansed of soft tissue, and stored in a 0.2% sodium azide solution at 4 °C for one month to inhibit microbial growth [23]. Upon the approval of the ethical committee of the faculty of dental medicine at the Saint-Joseph University of Beirut, Lebanon (FMD-221; ref.#USJ-2022-140), all these teeth were used for determining the research methodologies. For specimen preparation, the roots were sectioned and their crowns were embedded in gypsum, permitting the buccal enamel surface to be visible. Afterward, the enamel surface was abraded by means of an orthodontic grinder (Essencedental, Araraquara, SP, Brazil) until exposure to a flat medium dentin surface. The exposed dentin was later wet-ground with P320 silicon carbide sandpaper (SiC) for 1 min by means of a speed grinder-polisher (Buehler Ltd., Lake Bluff, IL, USA), under a water-cooling condition, at a motor speed of 70 rpm, for smear layer standardization and regulation. Then, the teeth were randomly distributed into four groups according to the adhesive systems. Subsequently, the specimens were divided into subgroups following the application modalities, totaling 16 subgroups (4 adhesive systems with 4 application techniques: AA, CV, CVM, and PA). Next, the evaporation of each applied adhesive system was carried out according to the instructions of the manufacturer.

All the bonding procedures were carried out by a single operator (RB) at room temperature and a constant relative humidity. After that, photo-activation was conducted during 20 s with a Light Emitting Diode (LED) multiwave light-curing unit Curing Pen (Eighteeth, Changzhou, China) using an irradiance of 1000 mW/cm^2^ [24,25,26], and resin composites (Reflectys, Itena Clinical, Paris, France) were applied in 3 increments of 2 mm each (A2 shade), and each layer was polymerized for 30 s with the same light-curing unit.

Following the bonding procedure and after immersion in distilled water at 37 °C for 24 h, the specimens were sectioned occluso-gingivally using a low-speed precision cutting machine (EXAKT Vertriebs GmbH, Norderstedt, Germany) into 1.0 mm × 1.0 mm composite-dentin beams. The μTBS was established in accordance with ISO/TS 11405, with the resin-composite fashioning the upper half of the beam and the underlying dentin forming the lower half of the beam. From each tooth, approximately fourteen beams were acquired. Half of the specimens (7 beams/tooth = 35 beams for 5 teeth) were evaluated after 24 h of aging in distilled water at 37 °C, while the other half (7 beams/tooth = 35 beams for 5 teeth) were stored at 37 °C in distilled water and evaluated after 6 months of aging [27].

### 2.3. Micro-Tensile Bond Strength Testing

Bonded resin-dentin beams were attached to a Geraldeli’s jig using cyanoacrylate glue (Zapit, Dental Ventures of North America, Corona, CA, USA), adapted to a universal testing machine (YLE GmbH Waldstraße, Bad König, Germany), and subjected to a tensile force until failure with a 1 mm/min crosshead speed and a 500 N load cell. Consequently, the cross-sectional area of each failed specimen was measured using a digital calliper with 0.01 mm of precision (Model CD-6BS Mitutoyo, Tokyo, Japan). By dividing the force at debonding [N] with the bonded surface area of the specimen [mm^2^], the μTBS value was calculated and expressed in MPa. For each tooth, the results obtained from the seven beams examined were averaged, and the mean attained was then used for statistical determinations (*n* = 5) [27].

### 2.4. Failure Mode Analysis

**Figure 2:**
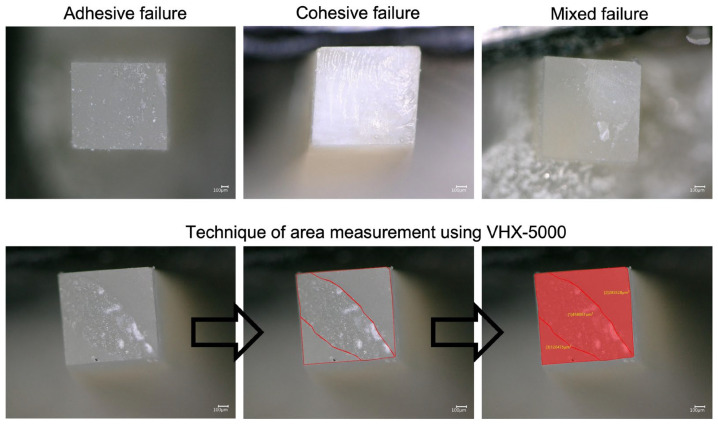
Representative images were obtained with an optical microscope at 150× magnification to recognize the failure mode in each specimen. The technique of area measurement was conducted using the VHX-5000.

All fractured portions were fixed to the aluminum stubs and observed under an optical numeric microscope (Keyence, Osaka, Japan) at 150× magnification to recognize the failure mode in each specimen. A VHX-5000 software was used to calculate the percentage of each area and to classify the type of failure as adhesive (the fracture site was within the adhesive), cohesive within composite, cohesive within dentin, or mixed (the fracture site extended into either the dentin or the resin composite). (Figure 2).

### 2.5. Scanning Electron Microscopy of Resin–Dentin Interface

Three resin-bonded beams, randomly selected from each subgroup (*n* = 3), were used for analysis of the composite-dentin interface morphology of specimens prepared as previously mentioned in the above test sessions. The specimen surfaces were etched with 37% phosphoric acid gel for 10 s, rinsed with distilled water for another 10 s, air-dried, and then immersed in a 2.5% sodium hypochlorite solution for 3 min. The specimens were finally washed with distilled water and placed in ascending grades of 25%, 50%, 75%, and 100% ethanol for sequential dehydration [28]. Thereafter, all specimens were immediately transferred for desiccation in a critical point drying machine (Balzers 030, Shimadzu, Kyoto, Japan). Specimens were then mounted on aluminum SEM stubs with conductive tape (double-sided carbon tape) and subsequently sputter coated for 120 s with a (20/80) ratio of gold-palladium using a sputtering device (Hummer JR, Technics, CA, USA). After gold sputtering, a Quanta 250 FEG SEM (FEI Company, Eindhoven, The Netherlands) operated at an accelerating voltage of 10 kV at different magnifications was used to analyze the specimens.

### 2.6. Adhesive Contact Angle

The adhesive CA to the dentin was measured with an optical tensiometer (Biolin Scientific, Espoo, Finland) following a sessile drop method. For each adhesive group, three dentinal surfaces were prepared to reach a flat dentin surface with P320 SiC. Then, a 5 μL drop of the adhesive system was placed on the prepared dentin surfaces. For this test, only the AA and PA techniques for OBFL and CSE were evaluated. In addition, for OBU and PBU, adhesives were applied directly (PA). By doing so, OBFL groups were subjected to acid etching for 15 s, rinsing for 15–30 s, and drying to obtain a moist dentin. Next, the dentin surfaces were subjected to primer application according to the respective modalities tested (AA of the primer for 20 s, then air-drying for 5 s; PA of the primer for 20 s, then air-drying for 5 s). Specimens of the CSE groups were subjected to acidic-primer application according to the respective modalities tested (AA of the acidic-primer for 20 s, then air-drying for 5 s; PA of the acidic-primer for 20 s, then air-drying for 5 s). For the OBU and PBU groups, since they were used in this study in a SE mode, the surfaces were not treated (only smear layer standardization by means of P320 SiC), and the adhesive system itself was placed on prepared dentin surfaces, besides the CA measurement was performed (PA of the adhesives, since an agitation could not be performed). An optical tensiometer (Biolin Scientific, Espoo, Finland) was used to evaluate the CA of the adhesive drop onto the material surface, which was measured after 10 s of contact by using a horizontal camera to track its profile. The experiment was repeated in triplicate (*n* = 3).

### 2.7. Statistical Analysis

SPSS (Version 29.0.1.0, IBM, Armonk, NY, USA) was used to perform statistical analysis. The data underwent analysis to examine the normal distribution and homogeneity of variance. The impact of the adhesive system and the application modalities on the μTBS to dentin was evaluated using a two-way analysis of variance (ANOVA). The bond strength was analyzed separately after 24 h and 6 months of aging. Data from the adhesive CA was subjected to a one-way ANOVA analysis including multiple comparisons (Bonferroni test), and a significance level of α = 0.05 was applied to all analyses.

## 3. Results

### 3.1. Micro-Tensile Bond Strength Testing

Table 2 summarizes the values obtained for the μTBS after 24 h aging according to the material and the technique used.

According to the ANOVA test, both factors and the interaction between them were significant (*p* < 0.001). The effect size for the factor adhesive was 0.5963, while the effect size for the factor technique was 0.2389; the effect size for the interaction between the factors was 0.3695. After 24 h, concerning the factor technique in the same adhesive group, PBU demonstrated significant differences between the techniques. Statistically higher bond strength was found for PBU in AA only when compared to the passive technique (*p* < 0.05), and PBU in PA showed statistically lower bond strength compared to the other techniques (*p* < 0.05). No significant difference was found between PBU in CV and PBU in CVM (*p* > 0.05). For the CSE, significant differences between the techniques were found. Statistically higher bond strength was found for CSE in CVM compared to the PA technique (*p* < 0.05), and CSE in PA showed statistically lower bond strength compared to the other techniques (*p* < 0.05). No significant difference was found between CSE in AA and CSE in CV (*p* > 0.05). Further, no significant differences were found between the used techniques among the other adhesive systems (OBU and OBFL) (*p* > 0.05). 

In addition, concerning the factor material, only the PA demonstrated significant differences between the tested materials. Statistically higher bond strengths were found for OBU and OBFL in PA compared to the other adhesives (CSE and PBU) (*p* < 0.05), while no significant difference was found between PBU and CSE as well as OBU and OBFL in PA (*p* > 0.05). No significant differences were found between the adhesive systems used among the other techniques (*p* > 0.05).

Table 3 summarizes the values obtained for the μTBS after 6 months of aging according to the material and the technique used. According to the two-way ANOVA analysis, the factor material was not significant (*p* = 0.153). On the other hand, the factor technique and the interaction between the factors were significant (*p* < 0.001). The effect size for the factor adhesive was 0.2017, while the effect size for the factor technique was 0.5289; the effect size for the interaction between the factors was 0.4773.

After 6 months of aging, concerning the factor technique in the same adhesive group, PBU, OBU, and CSE demonstrated significant differences between the techniques. Concerning PBU, statistically higher bond strength was found in AA compared to the other techniques (*p* < 0.05), while no significant difference was found between the bond strength values of the other techniques (*p* > 0.05).

AA and PA were significantly higher in the OBU adhesive (*p* < 0.05), compared to the lower values obtained in CV and CVM (*p* < 0.05). For the same adhesive, no significant difference was found between OBU in AA and PA, as well as between CV and CVM (*p* > 0.05).

Moreover, CSE in AA and CVM demonstrated statistically higher bond strength values compared to the other techniques (*p* < 0.05), while CSE in CV had statistically lower values compared to the other techniques (*p* < 0.05). No significant difference was found between CSE in AA and CVM (*p* > 0.05).

No significant differences were found between the techniques used for the OBFL adhesive (*p* > 0.05).

In addition, concerning the factor material, only when used with the CVM technique there were significant differences between the tested materials. A statistically higher bond strength was found for CSE and OBFL in CVM compared to the other adhesives (OBU and PBU) (*p* < 0.05), while no significant difference was found between OBFL and CSE, as well as OBU and PBU (*p* > 0.05). No significant differences were found between the adhesive systems used among the other techniques (*p* > 0.05).

Finally, Table 4 shows the means, standard deviations, and statistical analysis of the bond strength of all the tested adhesives with each technique at 24 h and 6 months of aging in distilled water.

Concerning CSE, only CV at 6 months demonstrated significantly lower bond strength compared to 24 h (*p* < 0.05), whereas no statistical differences were found for the other techniques between 24 h and 6 months (*p* > 0.05).

Concerning PBU, only CVM at 6 months revealed significantly lower bond strength compared to 24 h (*p* < 0.05), while no statistical differences were found for the other techniques between 24 h and 6 months (*p* > 0.05).

Concerning OBU, all the techniques at 6 months demonstrated significant lower bond strength compared to 24 h (*p* < 0.05), and the same observations were found for the OBFL, where all the techniques at 6 months revealed significant lower bond strength compared to 24 h (*p* < 0.05).

### 3.2. Failure Mode Analysis

The number of adhesive, mixed, and cohesive failures is described in Table 5.

For all the adhesive systems, most of the adhesive failures were adhesive or mixed fractures. On one hand, higher mixed failures were observed in samples that had higher bond strengths. On the other hand, higher adhesive failures in the samples revealed lower bond strengths among all the adhesive systems and application modes. 

### 3.3. Scanning Electron Microscopy of Resin–Dentin Interface

**Figure 3:**
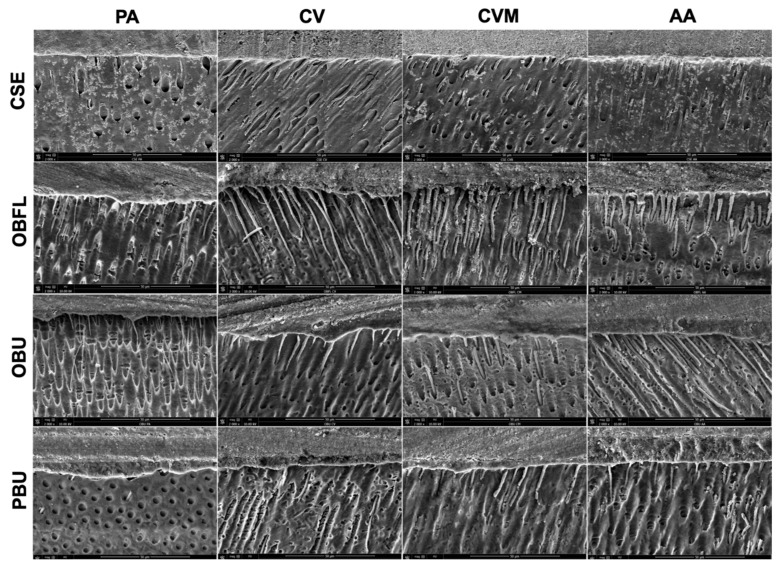
Representative scanning electron microscopy micrographs (×2000 magnification) reveal the adhesive layer and tag penetration of the different adhesive systems tested with various application modalities. Clearfil SE Bond (CSE); OptiBond FL (OBFL); OptiBond Universal (OBU); Prime&Bond Universal (PBU); Active application (AA); Compo-Vibes application (CV); Compo-Vibes modified application (CVM); and Passive application (PA).

SEM micrographs were taken in order to investigate the resin-dentin interface for the different adhesive systems with the different application modes (Figure 3). All the adhesives showed marked resin infiltration into dentinal tubules in AA among all the modalities tested. Less marked infiltrations were observed for the other application modes, regardless of the adhesive system. Different lengths and diameters of the resin tags were observed among all the techniques. According to the figures, dentin tubule orientation differs for each group. 

### 3.4. Adhesive Contact Angle

For this test, only the AA and PA techniques for OBFL and CSE were evaluated. Though OBU and PBU adhesives were applied directly (PA) to dentinal surfaces, an AA could not be performed.

For the PA mode, both universal adhesive systems (OBU and PBU) demonstrated statistically lower CA compared to the other systems (CSE and OBFL) (*p* < 0.05). No significant differences were found between OBU and PBU as well as between CSE and OBFL (*p* > 0.05).

**Figure 4:**
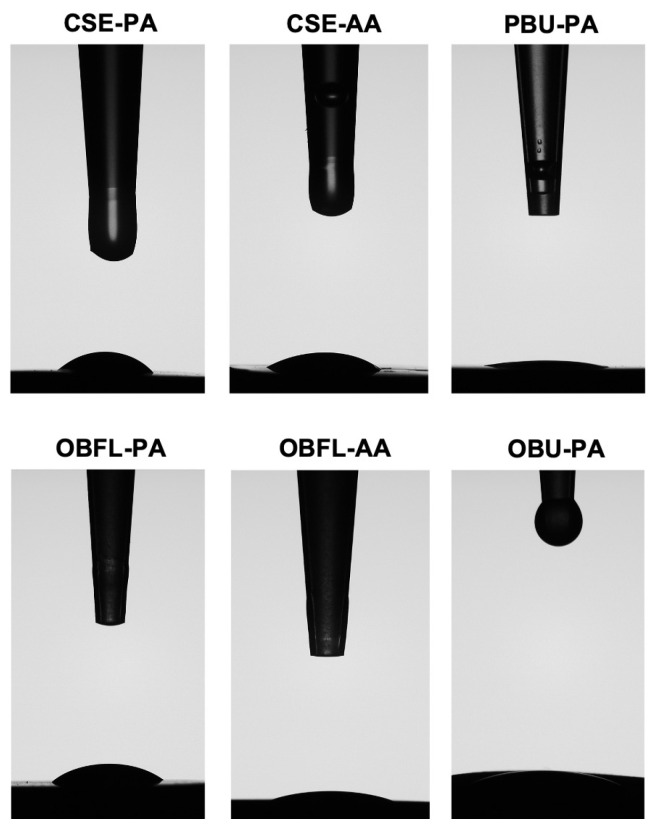
Representative images of the contact angles of different adhesives. All adhesives were measured after 10 s of contact by using a horizontal camera to track their profiles. The placement of the adhesive quantifies the intrinsic aptitude of the adhesive liquid to spread on a flat and solid dentinal substrate. Clearfil SE Bond (CSE); OptiBond FL (OBFL); OptiBond Universal (OBU); Prime&Bond Universal (PBU); Active application (AA); and Passive application (PA).

Concerning the AA mode, only CSE and OBFL were tested. In both groups, the AA demonstrated lower CA values compared to the same adhesives in PA (*p* < 0.05). No significant difference was found between OBFL and CSE in AA (*p* > 0.05) (Table 6 and Figure 4).

## 4. Discussion

In the present study, different application modalities (AA, CV, CVM, and PA) were implemented to enhance the bond performance of four adhesive systems to dentin. "Compo-Vibes”, as a novel device, was used to evaluate its effect on the bond strength of the different tested adhesive systems by using a brush or a micro-brush. Moreover, active and passive applications were included in order to investigate the effect of both modes on the bond strength of the different adhesive systems to dentin at 24 h and 6 months of aging. As far as the available reviewed literature is concerned, there have been no studies that evaluated the efficacy of this new device, ‘’Compo-Vibes’’. Most of the previous studies only incorporated active/passive/ultrasonic agitation of adhesive systems into the dental substrate [13,22]. Furthermore, some application modalities were evaluated to determine their influence on the wetting ability of adhesive systems. The results of the present study demonstrated that the material factor (adhesive system) and the application factor (application modality) influenced the bond strength values at 24 h and 6 months of aging. Further, the dentin wettability was affected by some adhesives under some modalities. Therefore, both null hypotheses tested in this study must be rejected. 

Concerning the technique factor, at 24 h, PBU and CSE presented a significant difference between their application modes (*p* < 0.05). AA and CVM demonstrated higher values for PBU and CSE, respectively, when compared to the other techniques, with a statistically higher bond strength found only when compared to the passive technique. All in all, the PA demonstrated lower values for both adhesives compared to the other techniques (Table 2).

According to precious research, the improvement in bond strength when an agitation (AA or CVM in this case) is used could be due to the deeper demineralization promoted by this technique, as fresh acidic resin monomers could be carried into the deeper areas [13,22]. Moreover, the agitation may increase solvent diffusion outward into the adhesive layer, allowing increased polymer cross-linking, degree of conversion, and other mechanical properties of the material [13,29]. The agitation action of adhesive systems (PBU and CSE) based on the hydrophobic nature of 10-MDP might have promoted deeper infiltration of MDP into the collagen network; therefore, their performance might differ when applied without agitation [6,12,30]. In this sense, as the mechanical properties of the adhesive layer increase with agitation, the quality of the adhesive interface increases, which could be translated into higher bond strength values and better conditioning of the substrate [13,30,31]. Passive-SE adhesives (CSE and PBU) application showed a noteworthy decrease in bond strength (Table 2). This could be elucidated by the higher amount of solvent inside the adhesive layer following the photopolymerization process. Accordingly, solvents presented after the light-curing increase the number of voids inside the HL and subsequently reduce the mechanical properties of the adhesive layer [29]. This might be the reason for the jeopardized immediate dentinal bond strength observed in this study for PBU and CSE in the PA modality.

Further, no significant differences were found between the used techniques among the other adhesive systems (OBU and OBFL) (*p* > 0.05). This proved the non-sensitivity of these adhesives (OBU and OBFL) to the modality applied (Table 2). The common feature between these adhesives relies on the fact that both materials are formulated with GPDM. Despite the fact that the literature regarding the effect of application modes on these adhesive systems is scarce, there are reports pointing out that the GPDM monomer can provide adequate micromechanical retention potential, bond strength, and durability similar to those of MDP-based materials [32,33]. It seems that the GPDM monomer after 24 h of bond strength to dentin is not sensitive to the application technique, and this feature could highlight the predictable results that this monomer provides and open the way for future research to be focused on this compound.

Regarding the factor material, at 24 h, only the PA demonstrated significant differences between the tested materials (*p* < 0.05). Higher bond strengths were found for OBU and OBFL in PA compared to the other adhesives (CSE and PBU). No significant difference was found between PBU and CSE, as well as OBU and OBFL in the PA (Table 2). Normally, the performance of an adhesive system is independent of the use of a single ingredient but rather of the overall balanced and optimal formulation, which contains a variety of components with different roles, including functional adhesive monomers [6,12,34]. PBU and CSE contain 10-MDP monomer, which was manufactured and patented by the Kuraray Noritake Dental Company [6,12]. Other manufacturers started implementing similar monomers, such as the GPDM monomer, which is found inside OBU and OBFL [12,35,36]. Technically, agitation action could have encouraged deeper penetration of the GPDM functional monomer into the collagen network, thus strengthening the adhesion of the resin composite to dentin [13,32], and no sensitivity to the passive or agitation modes was observed with this monomer [12,36], which was similar to the outcomes obtained in this study; hence, the performance of CSE and PBU immediately after 24 h might differ when applied without agitation (PA). Based on the information provided and the results of this study as well, passive applications should not be the application modality of choice for CSE and PBU. Worth is mentioning that the manufacturers of some specific materials (such as PBU in Table 1) claim to use their adhesive using an AA; therefore, clinicians must follow the right modality to ensure a proper bond [37]. Exclusively, the findings of this research propose that 10-MDP-based adhesive systems need to be applied with an agitation modality. However, 10-MDP-free adhesive systems could be applied in any tested modality (PA, AA, CV, and CVM). All in all, 10-MDP was sensitive to the PA modality, and GPDM was not sensitive to any modality applied.

With time, the HL is destroyed, and the strength of the dentin connection weakens due to the degradation of collagen fibrils and hydrophilic resin components. Specifically, this is due to the presence of collagen-degrading enzymes in dentin, such as matrix metalloproteinases (MMPs) and cysteine cathepsins [38]. The amplified awareness of the function of these enzymes in HL degradation has led to wide investigations targeting the prevention of collagenolytic activity at the resin-dentin interface [9,38,39]. In this manner, the samples tested were stored in distilled water at 37 °C in order to evaluate the effect of 6 months of aging on the dentinal bond strength of the different materials and techniques.

Furthermore, a significant difference in the bond strength after 6 months of water storage was observed for PBU, OBU, and CSE (*p* < 0.05). Concerning the technique factor, at 6 months, PBU had higher bond strength in AA compared to the other techniques (*p* < 0.05), while no significant difference was found between the bond strength values of the other techniques (*p* > 0.05) (Table 3). In order to elucidate the aforementioned outcome, one should keep in mind that PBU holds two functional monomers (10-MDP and dipentaerythritol pentaacrylate phosphate (PENTA)) that deliver an elevated bond strength; however, the presence of hydrophilic characteristics deteriorates the adhesion after long periods of storage, causing the highest reduction in bond strength, especially when deviated from the AA that is recommended by the manufacturer (Table 1). All in all, the mixture inside PBU might not favor stability, which explains the results obtained in this study [40].

AA and PA were significantly higher in the OBU adhesive (*p* < 0.05), compared to the lower values obtained in CV and CVM (*p* < 0.05). For the same adhesive, no significant difference was found between OBU in AA and PA, as well as between CV and CVM (*p* > 0.05) (Table 3). A possible explanation of this result could be the negative effect of micro-vibration by means of CV and CVM over the long term. The vibrational energy might negatively influence bond strength. Less difference in the depth of demineralization and resin infiltration might be estimated in SE adhesives [41,42], the penetration into the dentinal tubules is also lower than that of ER adhesives. Nonetheless, the restricted demineralization correlated to this type of adhesive system and the difficulty encountered by the adhesive in flowing through the smear layer may also contribute to the limited bond strengths when applied with CV and CVM. These data confirm the effects of Compo-vibes on the bond strengths of OBU used in SE mode with other agitation methods during their application, including continuous scrubbing, or PA.

Moreover, CSE in AA and CVM demonstrated statistically higher bond strength values compared to the other techniques (*p* < 0.05), while CSE in CV had statistically lower values compared to the other techniques (*p* < 0.05) (Table 3). CSE was the first SE adhesive system to incorporate 10-MDP monomer into both the bond and the primer [7,43]. Studies including CSE have established that MDP enables the formation of a stable chemical bond to the dentinal structure over time [44,45]. This could explain why this functional monomer is responsible for the higher bond strengths in AA and CVM. Moreover, it has been proven that the existence of hydroxy ethyl methacrylate (HEMA) inside the chemical composition of this adhesive system might compete with 10-MDP through bonding with the calcium of hydroxyapatite, which might be harmful to the chemical bond of MDP to the dentinal substrate. This could explain why the non-stability of the bond between the different components inside the two bottles of this system is due to the sensitivity of this specific bonding (CSE) to Compo-Vibes (Smile Line, St-Imier, Switzerland) by means of CV modality and PA. Although the manufacturers do not provide a detailed percentage of each constituent inside the bonding agents, it might be that the presence of diverse percentages of the 10-MDP functional monomer influences the degree of vulnerability of the adhesive system to the degradation process [46].

No significant difference was found between the used techniques among the adhesive system OBFL (*p* > 0.05) (Table 3). Regarding this finding, this adhesive was based on GPDM [33,47], which was not sensitive to the technique after 6 months, explaining the results. In addition, OBFL was the only ER adhesive system tested in this study, with non-variance of the bond strength between modalities observed after 24 h and even after 6 months of water storage. This might be related to the characteristics of this product. Previously, this adhesive was considered the golden standard material because of its good performance in immediate and long-term bond strength tests [47], which can be confirmed by this study. Despite the fact that the bond strength of OBFL after 6 months was not the highest when compared to the other adhesives, this was not significant. According to this data, the reason for its superior performance is related to the presence of GPDM, which can interact chemically with the hydroxyapatite [5], and the highly filled bonding resin layer (48 wt%) over the primed dental surfaces [48]. Considering this, it seems that the ER approach ensured that this adhesive technique is still a good option. 

Concerning the material factor, statistically higher bond strength was found for CSE and OBFL in CVM compared to the other adhesives (OBU and PBU) (Table 3). This is probably since these adhesives (OBU and PBU) were considered mild SE adhesives [6,12,49] compared to phosphoric acid (pH = 0.5) applied with OBFL or with CSE with a mild SE feature (pH around 2) [50]. Therefore, demineralization induced by UAs tested in this study did not reach deeper regions of the dentin through the micro-vibration produced by the micro-brush inserted into the Compo-vibes (Smile Line, St-Imier, Switzerland) device. Thus, a hypothesis could be formulated that the micro-vibration was not adequate to support the flow of the universal adhesive system into the free dentinal spaces and facilitate tag formation when compared to two-step SE and three-step ER. All in all, for long-term performance, the modality applied by clinicians using the CVM could be specific for some adhesives such as CSE and OBFL, which had the highest values after 6 months of using this modality. However, this is not true for the UAs tested in this study, which had relatively low values with this modality and the highest values with the active modality. So, the application of these specific UAs should be conducted in coherence with the AA for a better result; otherwise, deterioration of the adhesive could occur [51].

No significant differences were found between the used adhesive systems among the other techniques (AA, PA, and CV). This study’s results demonstrated that the AA mode is the most effective in increasing the bond strength throughout all the tested groups (Table 3). This shows that the active approach may be a more user-friendly alternative for the other application modalities in clinical practices. When a manual force is applied during the scrubbing of the adhesive systems on the dentin, the dentin surface acts as a sponge, and the collage matrix is compressed [30]. The compressed collagen network enlarges when the pressure is eased, and the penetration of the adhesive system into the collagen network may be enhanced [52]. Scrubbing action speeds up the evaporation of the solvent and the dispersion of water inside the adhesive systems as well [53], leading to the incorporation of a higher monomer rate inside the smear layer and underlying dentin [54]. These residual solvents might negatively influence the adhesive performance by reducing the polymerization effectiveness and altering the mechanical properties [29]. All in all, the application with an agitation modality benefits all the adhesive systems ranging from ER to SE and UAs at the bond strength level, consequently enhancing the properties of the adhesive layer. This application modality does not necessitate any extra steps. In addition, a previous systematic review and meta-analysis conducted by Hardan et al. showed that the use of a scrubbing modality improves both the immediate and the aged dentin bond strength [13]. This indicates that the AA was efficient between each adhesive, even after a long time, and that aging could not negatively affect the bonding performance when this modality is chosen. 

A novel adhesive application protocol based on the use of Compo-Vibes (Smile Line, St-Imier, Switzerland) by means of a brush (Smile Line, St-Imier, Switzerland) or micro-brush was incorporated in a study for the first time. Compo-Vibes (Smile Line, St-Imier, Switzerland) micro-vibrations by means of CV strengthened the bond to dentin for some adhesives, which can be justified by the enhanced monomer infiltration as well as prompting a better interaction with dentin. The release of the adhesive is triggered by the micro-vibration potential difference between the dentin surface and the adhesive. The CV modality provided similar performance as the AA but not the same bond strength values. A lower bond strength was observed for this modality. Moreover, this behavior shows that CV could not be recommended as an application technique for some adhesive systems. 

For the PA, the values of bond strength demonstrated no significant differences for the used adhesives (Table 3). This could be explained by the fact that some adhesive systems (including PBU and CSE) do not support the passive modality after a long period of storage. The incomplete solvent evaporation and a lower rate of monomer penetration inside dentin were key factors to understand when using this modality. Therefore, an incomplete polymerization with hydrogel retained-water formation might reduce the resin-dentin bond strength. This supports the non-difference between adhesives [55].

After 6 months of storage, CSE and PBU in one of the Compo-Vibes (Smile Line, St-Imier, Switzerland) modality (CV for CSE and CVM for PBU) demonstrated lower values compared to their values at 24 h (Table 4). No previous study was conducted on a Compo-Vibes (Smile Line, St. Imier, Switzerland) device. These results might be related to the sensitivity of 10-MDP to CV and CVM, respectively, for CSE and PBU, resulting in lower performance with aging. Micro-vibration might prevent the stability of the monomers inside these adhesives. Additionally, companies that manufacture these adhesives are advocates of AA for the PBU product, but the same might not be said for CSE adhesives; consequently, their performance might vary when applied with other modalities. Outlook studies are needed to test this hypothesis. 

OBU and OBFL demonstrated lower bond strength values after 6 months compared to the same bond at 24 h among all the application techniques due to the non-stability of the GPDM monomer with time (Table 4) [33]. It seemed that the GPDM monomer could go through hydrolysis at the highest rate in comparison with other monomers, such as the 10-MDP used in the formulation of the other adhesive systems. Its lower molecular weight and short-length spacer chains may compromise the chemical interaction with calcium and the dentin/enamel bonding performance [56]. In addition, dentin treated with GPDM appeared to be more hydrophilic than treated with 10-MDP [57], thus explaining the findings of this study.

Different failure modes were observed among all the groups after μTBS. Generally, the most observed failures were adhesive and mixed (Figure 2 and Table 5). This is in accordance with a previous study [58]. The cohesive failure in this study was rarely observed, and this failure could be due to an error in the composite application or a fragility in the dentin, which could generate a cohesive fracture in the composite or dentin structure. Moreover, it could be linked to voids or air bubbles in the composite structure [59]. Higher bond strength values showed higher mixed values in the present study. These results are in accordance with a previous manuscript [60]. The μTBS test protocol utilized a load force capable of passing throughout the dentinal substrate and the resin composite before attaining the adhesive interface, with consequent stress concentration at these sites [61], causing a high percentage of mixed failures. Further, this statement might also denote the good hybridization reached between the adhesive systems and the dentinal substrate [60]. After 6 months of water storage, failure analysis was mostly adhesive. This is linked to the aging of an adhesive layer, yielding more adhesive fracture when compared to the baseline (24 h) mode of failure [30].

In this study, the resin-dentin interface of the specimens was analyzed. SEM observations demonstrated a high number of resin tags among all the adhesive systems in AA compared to the other techniques (Figure 3). This could be due to the higher monomer infiltration presented with this modality into the branches of dentinal tubules [62]. In addition, this was possibly due to the elimination of non-infiltrated resin plugs comprised inside the tags by means of acids and bases used to dissolve all of the dentin from the resin [63]. Moreover, this could be associated with the fact that this application technique has been demonstrated to improve the interaction between the adhesive and the substrate, altering positively the biochemical characteristics of dentin and facilitating the penetration of the material within the inter- and peritubular zones [62].

The evaluation of the tag density, length, and size, as well as the thickness of the HL, could be influenced by the position of the dentinal tubules. Tubule diameters and densities increase from the dentin-enamel junction to the central dentin area. Therefore, all the SEM observations could be related to the investigated anatomical zone [64]. 

In addition, the wettability analysis evaluates resin and dentin interactions [65]. Thus, for optimum adhesion, a proper adhesive system with adequate spreading capacity and a low CA is essential [66]. Generally, the CA can be altered by the viscosity of a solution, heterogeneity, and surface roughness [67]. An ideal wettability may be attained when the free surface energy of the dental substrate is maximized and the adhesive system displays a lower CA [68]. Substrates with high wettability levels have a greater surface energy than the liquid’s surface tension [67]. The higher the viscosity of an adhesive system, the more difficult it is to wet a dental substrate [69]. In this study, CA values demonstrated that both UAs demonstrated lower values compared to the other systems in the PA (Table 6 and Figure 4). Dentin wetting was dramatically affected by acid etching. As it was pointed out in a previous work [70]. Since the acidity of UAs was lower than the other tested adhesives, lower wettability was clearly observed. Thus, promoting lower CA and higher spreading capacity.

Precisely, the AA mode could ameliorate the hydrophily of the dentinal surface among CSE and OBFL due to an increased spreading of the adhesive. This facilitated solvent evaporation and interaction with dentin [71]. Seemingly, to reduce the high viscosity of a monomer such as bisphenol A-glycidyl methacrylate (BIS-GMA) included in both adhesives (CSE and OBFL) tested, diluent monomers such as HEMA are included in the formulation. This could lower the viscosity of the solution, facilitating comonomer infiltration [69]. In addition, HEMA presented in these adhesives (CSE and OBFL) facilitates the wettability of AA because it is a hydrophilic humectant agent [72]. Presumably, both the dentin wetting by resin monomers and the spreading changed depending on the chemical characteristics of the adhesive. It could be hypothesized that the application modality might affect the surface wettability or directly influence the impregnation of adhesive monomers when using these systems [73].

However, one notable discovery presumed from this analysis was that there was no relation between the number of resin tags evaluated by SEM (Figure 3), the hydrophily of the different adhesive systems evaluated by CA (Figure 4), and the bond strength values measured by μTBS (Table 2, Table 3 and Table 4). OBU demonstrated lower CA than OBFL and CSE and markedly observed tags; in contrast, after 6 months of storage, its bond strength values were almost lower than those of CSE, which had a higher CA. In addition, PBU demonstrated fewer resin tags than OBFL; however, its bond strength values after 6 months were almost higher than OBFL values. These findings indicated that regardless of the adhesive layer and the length of the tags’ penetration into the dentinal tubules, adhesives with different chemical compositions, type, and qualities of the functional monomer contained in the materials’ composition may have an impact on the bonding performance in terms of degradation during prolonged storage in water [2]. Moreover, it was proven that there is no clear relationship between dentinal bond strength and surface wettability [74]. This can support the findings obtained in this study. In summary, bond strength, resin-tag penetration, and wettability were considered independent influencing factors when using each modality tested in this study with these dentinal bonding systems. 

Each bonding agent should be accompanied by the right modality during the application process. A previous survey showed that approximately 25% of dentists interviewed did not recall the appropriate application procedure of their bonding agent [37]. Therefore, it is possible that in dental practices, bonding agents are not applied based on the manufacturer’s instructions due to a lack of knowledge and time constraints in dentistry. A former study examined the effect of inaccurate use of six bonding agents on the bond strength [75] and found that the bond strength of resin composites to dentin was significantly weakened by deviations from the manufacturer’s protocol. Hence, this study proved that there are specific modalities in place for each adhesive system. 

Looking back at the advancements that adhesive systems have undergone throughout the previous 20 years, investigations into the actual improvements when it comes to technique sensitivity are crucial [62]. In this study, OBFL displayed the highest immediate and short-term values among all the tested adhesives. This shows that after 24 h, OBFL is not sensitive to any of the application modalities and supports previous studies’ claims of this adhesive’s noteworthy performance [12]. However, another study does not support the concept that this bonding agent is better than any other competitive products offered in the dental marketplace [76]. Having said that, practitioners and researchers alike should keep an eye on new literature findings. Some limitations could be addressed in this study. First of all, the number of materials tested was limited. Although representative brands of each of the types of materials available that are most currently used were included, it is well known that the in vitro performance of these adhesives is material-dependent, and more studies could be incorporated in future works. Moreover, for SEM observation, there were some samples that showed cracks and detached interfaces due to the high pressure of the SEM device. Moreover, the bond strength was only evaluated after 24 h and 6 months of aging in distilled water; more aging time or the use of other aging procedures such as thermocycling are desired in order to look for more signs of degradation of the adhesive interface.

## 5. Conclusions

Based on the findings of this study, it could be concluded that the bond strength could be influenced by both materials and application techniques. Following the right modality is considered an adhesive-dependent approach. It seems that the AA technique could be recommended as a gold standard for the application of an adhesive system to dentin. Plus, for long-term performance, the modality applied by clinicians using the CVM could be specific for some adhesives such as CSE and OBFL, which had the highest values after 6 months of using this modality. Moreover, the CV technique resulted in stability for PBU; consequently, their performance might vary when applied to other modalities. Future studies are needed to test this hypothesis.

## Figures and Tables

**Table 1:** Manufacturer and composition of the adhesives used.

Material	pH	Composition *	Manufacturer	Material Application	Active Application According to the Manufacturer Instructions
Prime&Bond Universal/Mild universal adhesive	pH = 2.5	10-MDP, PENTA, isopropanol, water, photoinitiator, bi- and multifunctional acrylate	Dentsply DeTrey GmbH, Konstanz, Germany	Adhesive was applied using the self-etch technique. One layer of adhesive was applied according to the modalities tested in this study for 20 s, and then mild air-blowing was carried out for 5 s. Adhesive was light irradiated for 20 s.	“Keep Prime&Bond Universal slightly agitated for 20 s”.
OptiBond Universal/Universal adhesive	pH = 2.5–3.0	Acetone, HEMA, GDMA, ethanol, GPDM	Kerr Co., Orange, CA, USA	Adhesive was applied using the self-etch technique. One layer of adhesive was applied according to the modalities tested in this study for 20 s, and then mild air-blowing was carried out for 5 s. Adhesive was light irradiated for 20 s.	“Apply a generous amount of OptiBond Universal adhesive to the enamel/dentin surface. Scrub the surface with a brushing motion for 20 s”.
OptiBond FL/Three-step etch-and-rinse adhesive	pH = Primer: 1.9; Bonding: 6.9	Etchant: 37.5% H_3_PO_4_ Primer: HEMA, GPDM, MMEP, water, ethanol, CQ and BHT Adhesive: Bis-GMA, HEMA, GDMA, CQ, and filler (fumed SiO_2_, barium aluminoborosilicat, Na_2_SiF_6_), coupling factor A174	Kerr Co., Orange, CA, USA	Etching for 15 s using a 37% phosphoric DENTOETCH acid (Itena Clinical, Paris, France). Rinsing with distilled water for 15–30 s. Air-drying for 15 s to obtain a moist dentin. One layer of primer and adhesive was applied according to the modalities tested in this study for 20 s. Mild air-blowing was carried out for 5 s after primer application and after adhesive application. In the case of the adhesive, this was light irradiated for 20 s.	“Apply material to the prepared enamel/dentin surfaces with a light scrubbing motion for 15 s”.
Clearfil SE Bond/Two-step self-etch adhesive	pH primer = 1.76 pH bond = 2	Primer: 10-MDP, HEMA, hydrophilic dimethacrylate, CQ, DEPT, water, ethanol. Bond: MDP, HEMA, bis-GMA, hydrophobic dimethacrylate, CQ, DEPT, silanized colloidal silica	Kuraray Noritake Dental Inc., Tokyo, Japan	One layer of acidic primer and adhesive was applied according to the modalities tested in this study for 20 s, and then mild air-blowing was carried out for 5 s after acidic primer and adhesive application. In the case of the adhesive, this was light irradiated for 20 s.	“Not specified”.

* Based on manufacturers’ MSDS. 10-MDP = 10-methacryloyloxydecyl dihydrogen phosphate; PENTA = dipentaerythritol pentaacrylate phosphate; HEMA = hydroxy ethyl methacrylate; GDMA = glycerol-dimethacrylate; GPDM = glycero-phosphate dimethacrylate; MMEP = methacryloyloxy-ethyl-dihydrogen phosphate; CQ = camphorquinone; BHT = butyl hydroxy toluene; Bis-GMA = bisphenol A-glycidyl methacrylate; DEPT = N,N-Diethyl-p-toluidine.

**Table 2:** Mean and standard deviation of the micro-tensile bond strength test (MPa) of the different application modalities for the adhesive system tested after 24 h aging.

Technique	CSE	OBFL	OBU	PBU
AA	^A^ 19.0 (3.2) ^ab^	^A^ 29.7 (5.2) ^a^	^A^ 28.3 (5.0) ^a^	^A^ 26.2 (8.9) ^a^
CV	^A^ 18.2 (6.1) ^ab^	^A^ 24.1 (6.8) ^a^	^A^ 25.1 (9.1) ^a^	^A^ 18 (5.6) ^ab^
CVM	^A^ 21.3 (3.9) ^a^	^A^ 27.3 (4.1) ^a^	^A^ 24.8 (2.8) ^a^	^A^ 17.5 (9.4) ^ab^
PA	^B^ 13.6 (3.8) ^b^	^A^ 30.3 (5.9) ^a^	^A^ 29.6 (5.2) ^a^	^B^ 10.9 (3.4) ^b^

Different uppercase letters indicate the presence of significant differences for each row (*p* < 0.05). Different lowercase letters indicate the presence of significant differences for each column (*p* < 0.05). Clearfil SE Bond (CSE); OptiBond FL (OBFL); OptiBond Universal (OBU); Prime&Bond Universal (PBU); Active application (AA); Compo-Vibes application (CV); Compo-Vibes modified application (CVM); and Passive application (PA).

**Table 3:** Mean and standard deviation of the micro-tensile bond strength test (MPa) of the different application modalities for the adhesive system tested after 6 months of aging.

Technique	CSE	OBFL	OBU	PBU
AA	^A^ 17.6 (3.8) ^a^	^A^ 14.9 (2) ^a^	^A^ 17.2 (6.6) ^a^	^A^ 21.1 (2.9) ^a^
CV	^A^ 9.5 (2.5) ^b^	^A^ 11.3 (3.4) ^a^	^A^ 7.9 (6.5) ^b^	^A^ 13.4 (6.8) ^b^
CVM	^A^ 18.3 (3.8) ^a^	^A^ 15.5 (2.1) ^a^	^B^ 7.9 (2.8) ^b^	^B^ 9.8 (3.4) ^b^
PA	^A^ 11.7 (6.3) ^ab^	^A^ 12.5 (1.3) ^a^	^A^ 12.4 (3.6) ^a^	^A^ 7.7 (2.8) ^b^

Different uppercase letters indicate the presence of significant differences for each row (*p* < 0.05). Different lowercase letters indicate the presence of significant differences for each column (*p* < 0.05). Clearfil SE Bond (CSE); OptiBond FL (OBFL); OptiBond Universal (OBU); Prime&Bond Universal (PBU); Active application (AA); Compo-Vibes application (CV); Compo-Vibes modified application (CVM); and Passive application (PA).

**Table 4:** Mean and standard deviation of the micro-tensile bond strength test (MPa) of the different application modalities as a function of the storing time for all the adhesive systems tested.

Technique/CSE	24 h	6 Months
AA	19.0 (3.2) ^A^	17.6 (3.8) ^A^
CV	18.2 (6.1) ^A^	9.5 (2.5) ^B^
CVM	21.3 (3.9) ^A^	18.3 (3.8) ^A^
PA	13.6 (3.8) ^A^	11.7 (6.3) ^A^
Technique/PBU	24 h	6 months
AA	26.2 (8.9) ^A^	21.1 (2.9) ^A^
CV	18 (5.6) ^A^	13.4 (6.8) ^A^
CVM	17.5 (9.4) ^A^	9.8 (3.4) ^B^
PA	10.9 (3.4) ^A^	7.7 (2.8) ^A^
Technique/OBU	24 h	6 months
AA	28.3 (5.0) ^A^	17.2 (6.6) ^B^
CV	25.1 (9.1) ^A^	7.9 (6.5) ^B^
CVM	24.8 (2.8) ^A^	7.9 (2.8) ^B^
PA	29.6 (5.2) ^A^	12.4 (3.6) ^B^
Technique/OBFL	24 h	6 months
AA	29.7 (5.2) ^A^	14.9 (2) ^B^
CV	24.1 (6.8) ^A^	11.3 (3.4) ^B^
CVM	27.3 (4.1) ^A^	15.5 (2.1) ^B^
PA	30.3 (5.9) ^A^	12.5 (1.3) ^B^

Different uppercase letters indicate the presence of significant differences for each row in each technique/bonding (*p* < 0.05). Clearfil SE Bond (CSE); OptiBond FL (OBFL); OptiBond Universal (OBU); Prime&Bond Universal (PBU); Active application (AA); Compo-Vibes application (CV); Compo-Vibes modified application (CVM); and Passive application (PA).

**Table 5:** Failure pattern analysis of the bonding agents evaluated after the micro-tensile bond strength test.

Technique/Material Fracture Mode (Adhesive/Mixed/Cohesive in Dentin or Resin)	CSE 24 h–6 Months	OBFL 24 h–6 Months	OBU 24 h–6 Months	PBU 24 h–6 Months
AA	16 adhesive/18 mixed/1 cohesive-18 adhesive/17 mixed/0 cohesive	11 adhesive/19 mixed/5 cohesive-17 adhesive/16 mixed/2 cohesive	11 adhesive/17 mixed/7 cohesive-17 adhesive/16 mixed/2 cohesive	11 adhesive/21 mixed/3 cohesive-16 adhesive/18 mixed/1 cohesive
CV	15 adhesive/17 mixed/3 cohesive-25 adhesive/10 mixed/0 cohesive	11 adhesive/22 mixed/2 cohesive-16 adhesive/17 mixed/2 cohesive	14 adhesive/19 mixed/2 cohesive-21 adhesive/10 mixed/4 cohesive	16 adhesive/17 mixed/2 cohesive-19 adhesive/15 mixed/1 cohesive
CVM	17 adhesive/15 mixed/3 cohesive-19 adhesive/16 mixed/0 cohesive	13 adhesive/22 mixed/0 cohesive-15 adhesive/15 mixed/5 cohesive	15 adhesive/18 mixed/2 cohesive-20 adhesive/14 mixed/1 cohesive	5 adhesive/28 mixed/2 cohesive-22 adhesive/12 mixed/1 cohesive
PA	20 adhesive/13 mixed/2 cohesive-21 adhesive/14 mixed/0 cohesive	14 adhesive/20 mixed/1 cohesive-17 adhesive/18 mixed/0 cohesive	13 adhesive/17 mixed/5 cohesive-15 adhesive/18 mixed/2 cohesive	23 adhesive/10 mixed/2 cohesive-27 adhesive/7 mixed/1 cohesive

Clearfil SE Bond (CSE); OptiBond FL (OBFL); OptiBond Universal (OBU); Prime&Bond Universal (PBU); Active application (AA); Compo-Vibes application (CV); Compo-Vibes modified application (CVM); and Passive application (PA).

**Table 6:** Contact angle (°) of 5 μL of adhesive droplet on the dentinal surfaces following passive or active applications.

	CSE	OBFL	OBU	PBU
PA (°)	^B^ 37 (1.4) ^a^	^B^ 32.5 (5.1) ^a^	^A^ 16.3 (0.1)	^A^ 12.9 (0.6)
AA (°)	^A^ 25.3 (1.8) ^b^	^A^ 23.4 (5.7) ^b^	X	X

Different uppercase letters indicate the presence of significant differences for each row (*p* < 0.05). Different lowercase letters indicate the presence of significant differences for each column (*p* < 0.05). Clearfil SE Bond (CSE); OptiBond FL (OBFL); OptiBond Universal (OBU); Prime&Bond Universal (PBU); Active application (AA); and Passive application (PA).

## Data Availability

The data presented in this study are available on reasonable request from the author (R.B.).
